# Developmental Trajectories and Sequential Analysis of Triadic Joint Attention

**DOI:** 10.1111/sjop.70096

**Published:** 2026-03-29

**Authors:** Tove Nilsson Gerholm, Petter Kallioinen, Tatjana von Rosen

**Affiliations:** ^1^ Department of Linguistics Stockholm University Stockholm Sweden; ^2^ Department of Statistics Stockholm University Stockholm Sweden

**Keywords:** child–parent interaction, developmental trajectories, multimodality, odds ratio, time‐window sequential analysis, triadic joint attention

## Abstract

Triadic joint attention (JA) refers to the shared focus of a child and an interlocutor on an object or event, accompanied by mutual awareness of this shared attention. Although JA is associated with early social interaction and later language development, its definitions and behavioral markers vary across studies and are often restricted to gaze and vocalizations, overlooking other communicative modalities. This longitudinal study followed 14 Swedish child–parent dyads during free play from 9 months to 3 years of age. Interactions were analyzed at both group and dyad levels. Vocal/verbal behavior, gesture, gaze, touch, and facial expression were annotated in detail. Time spent in JA was calculated across ages, and sequential analyses using odds ratios were conducted to examine how specific behaviors predicted the onset of JA. Joint attention increased steadily with age, accounting for 76% of interaction time at the group level by 3 years. Substantial variability was observed between dyads. Across ages, gaze combined with object‐directed action, neutral affect, and close physical proximity were the strongest predictors of JA, although their relative contributions varied across dyads. These findings highlight the importance of multimodal analyses and attention to individual variability for understanding the developmental role of joint attention. The methodological approach, time‐window sequential analysis, proved effective in identifying both group‐level patterns and the diversity between and within dyads' interactional styles. Moreover, analyses based on age measured in days indicated that age differences—even up to more than 1 month—played a minor role relative to this variability in dyadic interactional styles.

## Summary

- Large dyadic variability limits JA as a uniform predictor of language development.
- Group‐level predictors of triadic JA were gaze to object, proximity, neutral affect, and action gestures.
- Dyad‐level predictors of triadic JA were Failed JA and Gaze away preceding re‐engagement.
- Interactional patterns varied widely; some gestures were rare or absent.
- Sequential (odds ratio) analysis captured both general trends and individual variability.
- Interactional differences outweighed small age differences.

## 1 Introduction

The human capacity to coordinate gaze—encompassing gaze following, identification of a referent, and mutual acknowledgment of shared attention—is commonly defined as triadic joint attention (e.g., León 2021; Lasch et al. 2023). Since its introduction (Bruner 1974; Scaife and Bruner 1975), JA has been examined from multiple angles: the sequencing of its constituent behaviors (e.g., Woodward 2005); its association with later vocabulary and grammatical development (e.g., Carpenter et al. 1998; Akhtar and Tomasello 2000; Rollins and Snow 1998, Eriksson 2019); and its relevance to neurodevelopmental conditions such as autism (Dawson et al. 2004). Despite this extensive research, definitions and coding practices vary widely. A central point of disagreement concerns whether JA requires explicit mutual acknowledgment of shared attention (triadic JA) or whether coordinated attention without such acknowledgment (dyadic JA) is sufficient.

These divergent operationalizations may contribute to inconsistent findings regarding JA's predictive value for later language and cognitive outcomes (Akhtar and Gernsbacher 2007; Astor and Gredebäck 2022). Although both dyadic and triadic JA emphasize shared attention, the behaviors through which JA is initiated, maintained, and confirmed – eye gaze, vocalizations, gestures, facial expressions, or touch – are seldom described with precision. An additional challenge arises from substantial individual variability in developmental trajectories. Because milestones such as first words vary widely across children, analyses based solely on chronological age may obscure meaningful differences in how children engage in JA, potentially weakening associations between JA frequency or responsiveness and later outcomes.

To better understand how caregiver‐child dyads engage in JA, and which behaviors predict the emergence of triadic JA at both group and individual levels, a fine‐grained, behavior‐by‐behavior analysis is required. The present study addresses this need by examining triadic JA sequences in 14 caregiver‐child dyads observed longitudinally across eight sessions from 9 to 36 months of age.

### 1.1 Joint Attention

#### 1.1.1 Terminology and Definitions

Since Bakeman and Adamson's (1984) seminal investigation into how infants coordinate gaze behavior with mothers and with peers, a range of terms have been used to describe similar phenomena. These include “coordinated joint attention” (Bakeman and Adamson 1984), “joint visual attention” (Scaife and Bruner 1975), “triadic attention” (de Barbaro et al. 2016; Striano and Stahl 2005), “shared attention” (Deák et al. 2017; Siposová and Carpenter 2019), “coordinated visual attention” (Yu and Smith 2017), and “coordinated attention” (Chen et al. 2019). As Gabouer and Bortfeld (2021) observe, this terminological variation is not merely cosmetic; it has substantive implications, particularly given that the term joint attention is frequently employed as an umbrella concept and has clinical relevance—for example, in the assessment and diagnosis of autism spectrum disorders (Kasari et al. 2012).

The choice of terminology often reflects differing theoretical orientations. These include debates over whether JA should be conceptualized primarily as a social‐cognitive capacity or as an associative process (see Gabouer and Bortfeld 2021, for discussion). Furthermore, these theoretical perspectives influence which behaviors, beyond gaze coordination, are included in analyses, as well as the assumed developmental function of JA—whether it primarily facilitates social interaction or language acquisition.

When conceptualizing joint attention as a stepwise developmental process, the initial interactional move typically involves the shared direction of gaze toward an object (Brennan et al. 2008). To qualify as dyadic joint attention, however, participants must also engage in communicative behaviors related to the jointly attended object, such as verbalizations, deictic gestures, or pointing (Oates and Grayson 2004). Triadic joint attention—the focus of the present study—represents a more advanced stage of social engagement, requiring explicit confirmation of shared attention. This is typically manifested through a gaze directed toward the interlocutor, thereby signaling mutual awareness of a shared referent (e.g., “I know that you know we are both attending to the same object or event”; Reddy 2005). The rationale for this acknowledgment behavior in triadic joint attention is that, in its absence, the participants' actions may remain parallel rather than truly shared—each may touch or look at the same toy, yet without attending to one another. This ambiguity is particularly relevant in laboratory settings, where parent and child are isolated in a room with toys and cameras, a context that inevitably increases the likelihood that both focus on the same objects.

#### 1.1.2 Development and Frequency of JA

In their study of 28 children aged between 6 and 18 months interacting either with their mother or a peer, Bakeman and Adamson (1984) found that coordinated joint attention (i.e., triadic JA) constituted approximately 2% of interactions at 6 and 9 months of age. The proportion of coordinated JA increased with age, doubling between 15 and 18 months. Moreover, children engaged in joint attention for longer durations when interacting with their mother compared to a peer, suggesting that the early emergence of successful JA is influenced by caregiver scaffolding and responsiveness. Additional research indicates that children are more likely to engage in JA when caregivers refer to objects already within the child's visual focus, rather than to objects outside of it (Rollins 2003). Supporting this, Yu and Smith (2013) found that a child's gaze toward objects had greater predictive value for successful interaction in an experimental setting than did more general gaze behaviors, such as mutual gaze between parent and child.

Turning to non‐WEIRD contexts, Mastin and Vogt (2016) investigated a sample of rural and urban Mozambican children aged 1;2 years, finding that coordinated joint attention (triadic JA) accounted for 17%–18% of interaction time. Similarly, Childers et al. (2007) reported that eight children aged 1;2 to 2;7 years in a rural Nigerian village spent 26% of their interaction time engaged in coordinated triadic JA.

### 1.2 Multimodal Interactions and JA

Although not required for the formal definition of triadic joint attention, pointing is widely regarded as a core component of JA, social understanding, and later language development (e.g., Donnellan et al. 2020; Woodward 2005; Colonnesi et al. 2010; Brooks and Meltzoff 2005). A meta‐analysis by Colonnesi et al. (2010) identified declarative—though not imperative—pointing as a strong predictor of language acquisition, with the association strengthening with age. As with JA more broadly, however, cultural variability has been noted, with studies in non‐Western contexts reporting divergent patterns (Blake et al. 2003; Salomo and Liszkowski 2013).

Touch has also been proposed as an early precursor of JA, given its developmental primacy and its role in communication among non‐human primates (Botero 2016). Tactile exploration has been linked to early word learning, particularly for objects accessible to both touch and vision (Smith and Yu 2008), and experimental work suggests that touch facilitates infants' learning of body‐related vocabulary (Seidl et al. 2015). Within JA episodes, touch frequently serves as an initiator, especially among deaf or hard‐of‐hearing children and their caregivers (Koester and Lahti‐Harper 2010; Loots and Devisé 2003; Gabouer et al. 2020).

Facial expressions may also support JA. Parents commonly pair pointing with smiling when directing infants' attention (Leavens et al. 2014), potentially enhancing the reward value of the interaction. Emotional expressions influence gaze behavior more generally, with happy faces eliciting faster gaze shifts than neutral or angry faces (Hori et al. 2005), consistent with broader evidence for a “happy advantage” in social attention and learning (e.g., Flom and Pick 2005). Whether such effects directly shape JA, however, remains unresolved.

### 1.3 Individual Differences

Children vary substantially in their language acquisition trajectories (e.g., Bornstein et al. 2014; Bornstein et al. 2016). Nevertheless, research in first language acquisition typically relies on group‐level analyses in which chronological age serves as the primary organizing variable. Given the considerable individual variation observed in early development, age often functions more as a confounding factor than an explanatory one. This may help account for the many studies—including those on joint attention—that, despite employing comparable methodologies, assessment tasks, age ranges, and research questions, nonetheless report divergent findings (e.g., Markus et al. 2001; De Schuymer et al. 2011; Astor and Gredebäck 2022; Mundy et al. 2007; Slaughter and McConnell 2003; Çetinçelik et al. 2021). Such inconsistencies are further compounded by the wide age ranges (in days) often present within nominally homogeneous age groups.

To determine whether and how individual differences shape research outcomes, fine‐grained analyses based on smaller samples are essential. The present study represents one such effort.

### 1.4 The Present Study

Given the heterogeneous definitions and operationalizations of joint attention—and the inconsistent findings regarding its relation to later language abilities—we set out to examine triadic JA in greater depth. We focused specifically on triadic rather than dyadic JA because the former offers clearer annotation criteria, including explicit indicators of shared gaze or vocalization directed toward a mutual object of attention.

By examining triadic JA longitudinally, we aimed to compare group‐level patterns with the individual trajectories of the eight focal dyads, thereby offering a more nuanced understanding of how a presumed universal developmental phenomenon—JA in Western populations—may vary across children and across age.

To delineate the developmental profile of triadic JA, we formulated two research questions:

- What are the developmental characteristics of triadic JA in this dataset, both across age groups and within individual dyads?
- Which behaviors predict triadic JA over time, at both the group level and the dyadic level?

Through RQ1, we sought to situate our findings in relation to the existing literature, including potential differences arising from data collection contexts (e.g., home vs. laboratory). We also investigated the extent to which dyads vary in their reliance on JA, in order to better assess the generalizability of JA as a construct within research on language acquisition. Furthermore, we used time‐window sequential analysis to identify behaviors that predict the onset of triadic JA sequences (RQ2). Such predictors may differ across developmental stages and between dyads, offering insight into which interactional behaviors most reliably initiate mutual engagement.

## 2 Materials and Methods

### 2.1 Participants and Data Collection

The data analyzed in this study were drawn from the MINT project (The Role of Interaction and Parental Input in the Language Acquisition Process; MAW 2001.0070; VR 2018‐01135; Nilsson Gerholm 2025). Fourteen child–parent dyads (seven girls) were selected based on the completeness of behavioral annotations. The participating parent was either the mother or the father.

All children had Swedish as their first language, with one child also exposed to an additional language. At 2 years of age, all children were developing typically, all had begun attending preschool (15–40 h per week), seven had at least one sibling, and all lived in two‐parent households. The families' combined annual incomes were within the middle to upper range, and all but one child had at least one parent with a university‐level education (for detailed description see Supporting Information S1a).

**FIGURE 1:**
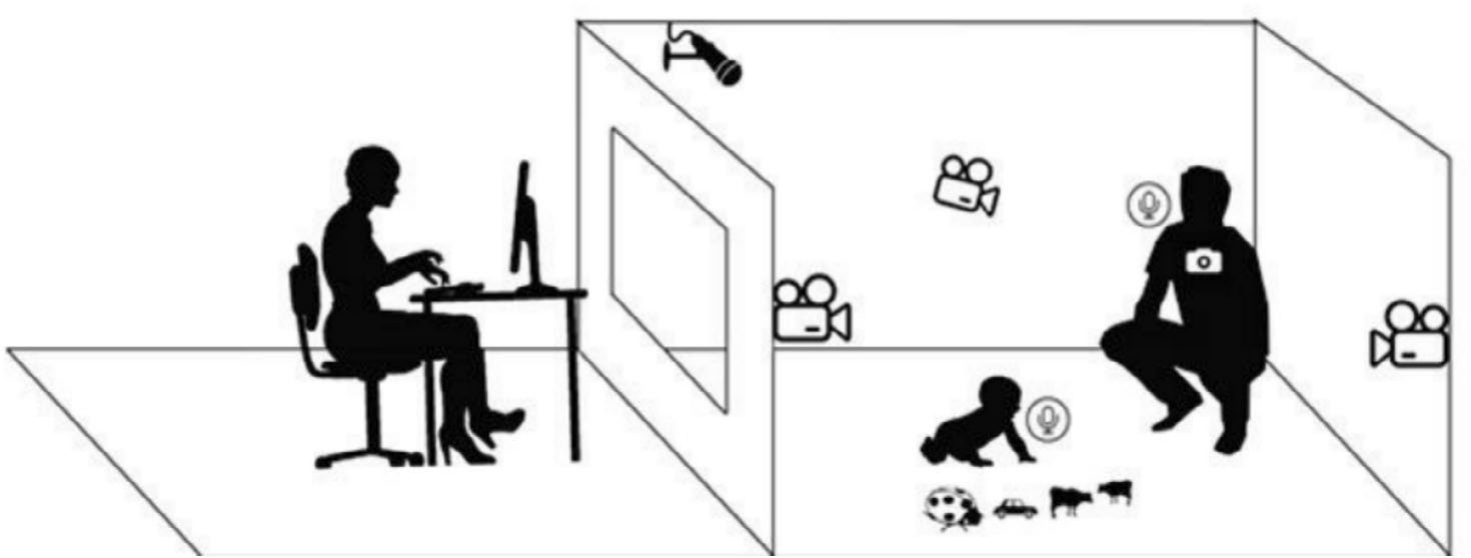
Interaction lab set‐up.

Recordings were conducted in a small, child‐friendly room equipped with three stationary video cameras and one body‐mounted InAction camera worn by the parent (see Figure 1). Both the child and the parent wore individual microphones. The room contained a soft carpet, pillows, and a selection of age‐appropriate toys. Parents were instructed to engage in play as they normally would, using the available toys. Each session lasted 10–15 min. As triadic joint attention typically emerges around 9–10 months of age (Tomasello 1995; Adamson and McArthur 1995), the sample for this study includes recordings beginning at 9 months.

### 2.2 Data and Annotation Procedures

Audio‐visual data from eight free‐play sessions involving 14 parent–child dyads were analyzed. Six sessions were recorded quarterly between the ages of 9 and 24 months, and two additional sessions at 30 and 36 months. Recordings were annotated by trained research assistants and the first author using ELAN software (Wittenburg et al. 2006), following a standardized transcription protocol (see Supporting Information S2).

For each dyad, the onset and offset times of vocalizations/verbalizations, gaze, gestures, facial expressions, touch were annotated, followed by annotation of successful and unsuccessful triadic joint attention.

To validate the transcriptions and annotations, 5 min of 25% of the dataset were independently annotated by a second observer. This sample included four randomly selected files each from the Vocal, Gesture, Gaze, and Joint Attention tiers, and six each from Touch and FacialExpression/Mood, representing different child ages.

The Kappa and Staccato tools in ELAN were not used, as they do not adequately capture agreement in relation to the present study's analytical focus on temporal duration and frequency. Instead, the two versions of each annotated file were compared on a separate tier with three possible outcomes: Complete agreement, Partial agreement, or No agreement (see Supporting Information S4).

Complete agreement was defined as identical or near‐identical timestamps and content (minor differences in milliseconds or spelling were disregarded). Partial agreement was used when timestamps differed slightly without affecting duration measures, or when segmentation differences did not alter interpretation (e.g., one annotator dividing an utterance into clauses, the other merging them). No agreement was used when timestamps differed substantially or when annotations diverged categorically (e.g., one coding a gesture as Deictic, the other as Emblem).

Agreement was calculated as the combined percentage of Complete and Partial agreements, yielding: Vocalizations 95.8% (79.3%–100%), Gestures 81.1% (76.5%–93%), Gaze 86.4% (77.5%–93.3%), Touch 67.3% (54.5%–83.3%), FacialExpression 74.8% (72.7%–81%), and Joint Attention 87.5% (84.3%–91.2%). Agreement was satisfactory to excellent except for Touch and FacialExpression, where reliability was reduced by the number of fine‐grained subcategories (e.g., press, push, pat for Touch; happy, excited, surprised for FacialExpression/Mood).

To improve consistency, composite categories were introduced: Comfort, Stimulate, and Action for Touch; Positive, Neutral, and Negative for FacialExpression. Re‐annotation of four additional files (two per category) increased agreement to 84.2% (77.4%–91%) for Touch and 89.0% (84.2%–94.1%) for FacialExpression. These composite categories were used in all subsequent analyses.

#### 2.2.1 Behavioral Coding

Table 1 presents an overview of variables and acronyms. Child vocalizations at 9 months of age were annotated using Controlled Vocabulary with the categories expressive (e.g., crying, laughter), explorative (e.g., cooing), babbling, and utterances (proto words and words). From 12 months of age, all child vocalizations were coded orthographically. In the analyses all child vocalizations were included in the variable Child_Vocal. This variable thus includes not only words and protowords but also babbling, laughter, and communicative sounds like cooing and grunting. Non‐communicative sounds such as sneezes, coughs and panting were not transcribed. Parent vocalizations (Parent_Vocal) were primarily verbal and transcribed orthographically but laughter and non‐verbal sounds of a communicative nature (such as mimicking the child's babbling) were included.

**TABLE 1:** Overview of variables and composites.

Modalities	Acronyms	Explanations
Gaze	CG_object, PG_object	Gaze object
	CG_away, PG_away	Gaze away
	PG_child	Gaze child
	CG_parent	Gaze parent
Gesture	CGe_Action, PGe_Action	Gesture action: play
	CGe_Deictic, PGe_Deictic	Deictic: gesture pointing
	CGe_Emblematic, PGe_Emblematic	
	CGe_Emphatic, PGe_Emphatic	
	CGe_Iconic, PGe_Iconic	
	CGe_show/offer, PGe_show/offer	
Touch	CT_Action, PT_Action	Action_T: hit, kick, play, correction
	CT_Comfort, PT_Comfort	Comfort: stroke, hold, lift, rest, pat, hug, kiss
	CT_Stimulate, PT_Stimulate	Stimulate: pull, push, press, poke, rub, scratch
Mood	CM_Neutral, PM_Neutral	Neutral mood
	CM_Positive, PM_Positive	Positive mood: joy, anticipation, drama, interest
		e , surprise, excitement
	CM_Negative, PM_Negative	Negative mood: anger, fear, concern, frustration—
		irritated, sad
Vocalization	C_Vocal	Vocal: babbling, explorative, expressive, utterance—
	P_Vocal	Other

*Note:* The first letter in acronyms: P stands for Parent, and C stands for Child. For example, CG_away: Child Gaze away.

Gaze behavior was coded continuously and categorized as directed at the interlocutor (CGp/PGc), at objects (CGo/PGo), away (CGa/PGa), or out of view (excluded from analysis). Gestures (CGe/PGe) included deictic, iconic, emblematic, emphatic, show/offer, and two additional tags capturing object‐oriented activities: action (e.g., a child driving a toy car on the floor) and handle object (e.g., exploring an object with hands or mouth).

Touch was coded by function (Mantis et al. 2014) as comfort (e.g., patting), stimulation (e.g., poking), or action (e.g., hitting). Facial expression was annotated based on facial movements regardless of concurrent speech or actions (sound off during annotation). This was in order not to be misled by the intonation and speech bias. Facial Expression categories were collapsed into CM/PM_positive, CM/PM_negative, and CM/PM_neutral.

To capture the multimodal structure of JA and to analyze triadic JA alongside its component behaviors (e.g., Child Gaze object, Parent Gaze object, Child Gaze parent, Parent Gaze child), we additionally constructed composite variables. Definitions of these composites are provided in Table 2.

**TABLE 2:** The definition of the composite variables.

Composite variable	Behaviors included	Composite variable	Behaviors included
SGo SGoPV CGoPV PGoPV	CG_object *∩* PG_object SGo *∩* PV_utterance CG_object*∩* PV_utterance PG_object *∩* PV_utterance	C_Vocal CM_Positive CM_Negative CT_Comfort	CV (babbling *∪* explorative *∪* expressive *∪* utterance *∪* other) Child Mood (joy *∪* anticipation *∪* drama *∪* excitement *∪* interest *∪* surprise) Child Mood (anger *∪* fear *∪* concern *∪* frustration *∪* irritated *∪* sad) Child Touch (stroke *∪* hold *∪* lift *∪* pat *∪* rest *∪* hug *∪* kiss)

*Note:* The symbol ∩ stands for the intersections of variables, seconds with overlap of variables was extracted to form a composite; the symbol ∪ stands for the unions of variables, seconds with activity in any of the variables was added to form a composite.

#### 2.2.2 Triadic Joint Attention Annotation

Triadic joint attention was identified following Tomasello and colleagues (Tomasello and Todd 1983; Tomasello and Farrar 1986). An episode required: (i) initiation by one partner around an object or event; (ii) mutual focus sustained for at least 3 s; and (iii) overt indication of shared attention, such as gaze shifts, gestures, or verbal acknowledgment. Episodes were further classified by initiator: child‐initiated (CJA) or parent‐initiated (PJA).

Failed joint attention (Failed_JA) was defined as instances where an initiation occurred but mutual acknowledgment did not follow (i.e., criteria (i) and possibly (ii) were met, but not (iii); (cf., Eriksson 2019)).

Joint attention was annotated after all other annotation tiers had been completed. The annotator first identified, by reviewing the video recordings, sequences in which the child and the parent were simultaneously focused on the same object or event. The annotator then consulted the corresponding transcripts to determine the behavior that appeared to initiate the joint engagement. The JA tag was maintained until one of the interactants terminated the engagement, except in cases of brief gaze aversion, which was not considered termination if gaze was subsequently returned to the interlocutor.

Failed joint attention (Failed‐JA) was annotated when one interactant made a clear attempt to establish engagement—typically through vocalization, gaze, or gesture—but the other interactant did not respond. Failed‐JA attempts were annotated for the duration of the initiating behavior. Examples of both successful and failed JA initiations are provided in Supporting Information S3.

### 2.3 Data Preparation

The analyses draw on interactions ranging from 6.15 to 18.15 min (M = 10.52, SD = 2.13), recorded with only the parent and child present. ELAN annotations were exported (see Supporting Information S5 for code), and each behavior was coded as a binary value for every second of the session. There is no golden rule for choosing the length of the time‐window (Chorney et al. 2010). The recommendation is to use time window durations that make sense concerning the nature of the data. In our study, the rate at which behaviors are occurring made us chose a short duration for the time‐window (1 s). Behavioral shifts in interaction are rapid and short lived, like gaze shifts, pointing, and facial expressions. A possible draw back with the short time window is that the distinction between behaviors predicting JA and behaviors initiating JA is hard to establish. However, this problem remains regardless time window, as behaviors with potentially longer duration, such as comforting touch, is as likely to both be predictive of a JA and remain as part of the JA it predicted. For comparability across recordings, analyses were based on the proportion of session time in which each behavior occurred in relation to the total duration of the session. In total, 55 variables were annotated; 36 were retained for the OR analysis, with infrequent behaviors excluded. Table 1 presents an overview of all annotated behaviors, grouped by modality and subtype.

Although we aimed to conduct the recordings as close as possible to each child's target age (9, 12, 15 months, etc.), practical circumstances such as family vacations, illness, and logistical constraints occasionally prevented exact scheduling. The deviations (in days) from the intended age milestones were as follows: 9 months (M = 10.7; range = −13 to +53), 12 months (M = 4.9; range = −12 to +10), 15 months (M = 4.9; range = −11 to +10), 18 months (M = 8.4; range = −11 to +32), 21 months (M = 7.5; range = −12 to +26), 24 months (M = 7.2; range = −14 to +20), 30 months (M = 5.7; range = −21 to +8), and 36 months (M = 7.9; range = −16 to +9). Further details are provided in Supporting Information S1b.

### 2.4 Statistical Analysis

The statistical analyses were performed with IBM SPSS statistics, version 29 (IBM Corp., Armonk, N.Y., USA) and SAS Studio (SAS On Demand for Academics).

We present the trajectories of JA development with descriptive statistics and correlations. Time‐window sequential analysis was used to explore the temporal relationship between behaviors, and calculate the predictive value of specific behaviors or behavioral clusters for a JA‐episode. In time‐window analysis the data is organized in a 2 × 2 contingency table, and the corresponding Odds Ratio is calculated (see Yoder and Tapp 2004). The odds ratios above 1 indicate that JA is more likely to occur relative to a given behavior X.

The Odds Ratio can be transformed into another index of effect size, Yule's Q that ranges from −1 to +1, Q = (OR—1)/(OR + 1). If Q = 0, there is no relationship between the JA and behavior X.

As behaviors could predict JA both as independent variables and as part of a composite, no distinction between discrete and composite behaviors was made.

## 3 Results

### 3.1 Amount of Triadic JA Over Time

To investigate the amount and development of JA, we compared the JA‐sequences following the definition of triadic JA with Failed‐JA, and divided the successful JA‐sequences into child‐ and parent‐initiated respectively (see Section 2.2.2).

**FIGURE 2:**
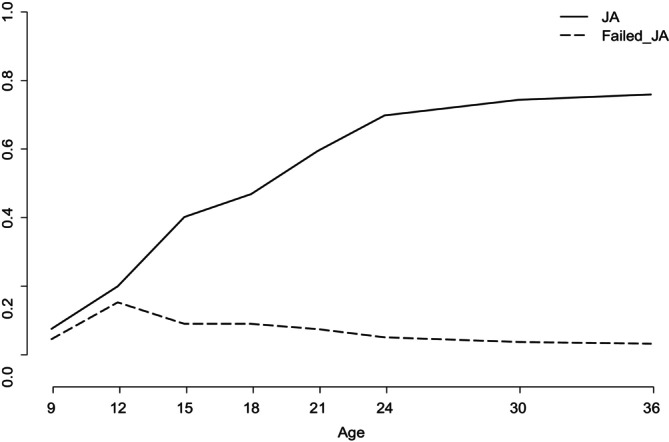
Average time that parents and children (*n* = 14) established JA, and failed to establish JA over age. The Y‐axis shows the percentage of time spent in the behavior. The X‐axis shows the age in months.

**FIGURE 3:**
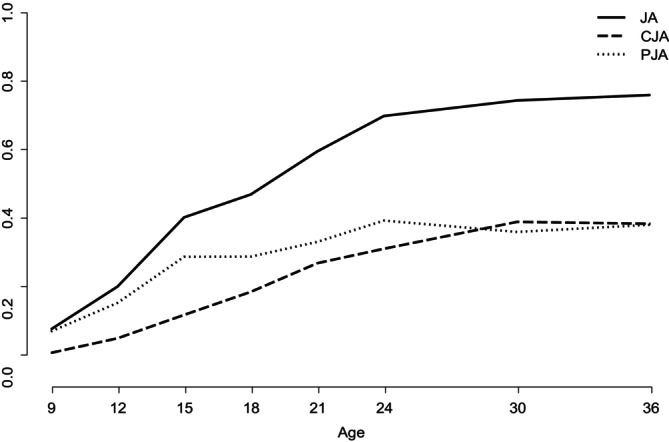
Average percentage of time in either a parent‐ or a child‐initiated JA (PJA/CJA) during the child's first 3 years. The *Y*‐axis shows the percentage of time spent in the behavior. The *X*‐axis shows the age in months.

The amount of time that the children spent in JA (Figure 2) increased steadily from 7.5% at 9 months of age to 76% at 36 months of age. As illustrated in Figure 3, JA was more frequent as parent‐initiated than child‐initiated throughout the period of 9–30 months.

Investigating the length of JA and the frequencies of CJA and PJA (Table 3), we see that the mean length of JA‐sequences increases from 13.28 s at 9 months of age, to 37.01 s at 36 months of age, while frequency of JA increases more dramatically, from 96 (total) at 9 months of age, to 366 (total) at 36 months of age. Apart from the recording at 30 months of age, parent‐initiated JA‐sequences are longer throughout the recording period. While both measures show an expected increase over age, frequency accounts for the largest difference (for min, max for CJA and PJA over dyads and ages, see Supporting Information S6). Child‐initiated JA (CJA) further goes from 11 sequences at 9 months of age (5 of the children could initiate JA at this age), to 193 JA‐sequences at 36 months of age. Frequency of parent‐initiated JA was more evenly distributed across age with the lowest amount at 9 months. From 21 months of age children and parents initiate approximately the same number of JA.

**TABLE 3:** Mean length of JA divided into CJA, PJA, and Total, and the frequencies of CJA, PJA, and Total JA over age.

Age	CJA mean length	PJA mean length	JA_total mean length	Age	CJA mean length	PJA mean length	JA_total mean length
9	3.16	10.11	13.28	21	11.85	18.19	30.03
12	5.21	10.78	16.00	24	15.66	19.68	35.33
15	9.12	13.93	23.05	30	18.43	18.06	36.49
18	11.35	14.95	26.31	36	17.37	19.64	37.01

Age	Number of CJA	Number of PJA	JA_total	Age	Number of CJA	Number of PJA	JA_total
9	11	85	96	21	166	154	320
12	41	105	146	24	167	170	337
15	99	165	264	30	180	165	345
18	129	153	282	36	193	173	366

#### 3.1.1 Differences Across Dyads and Individuals

**FIGURE 4:**
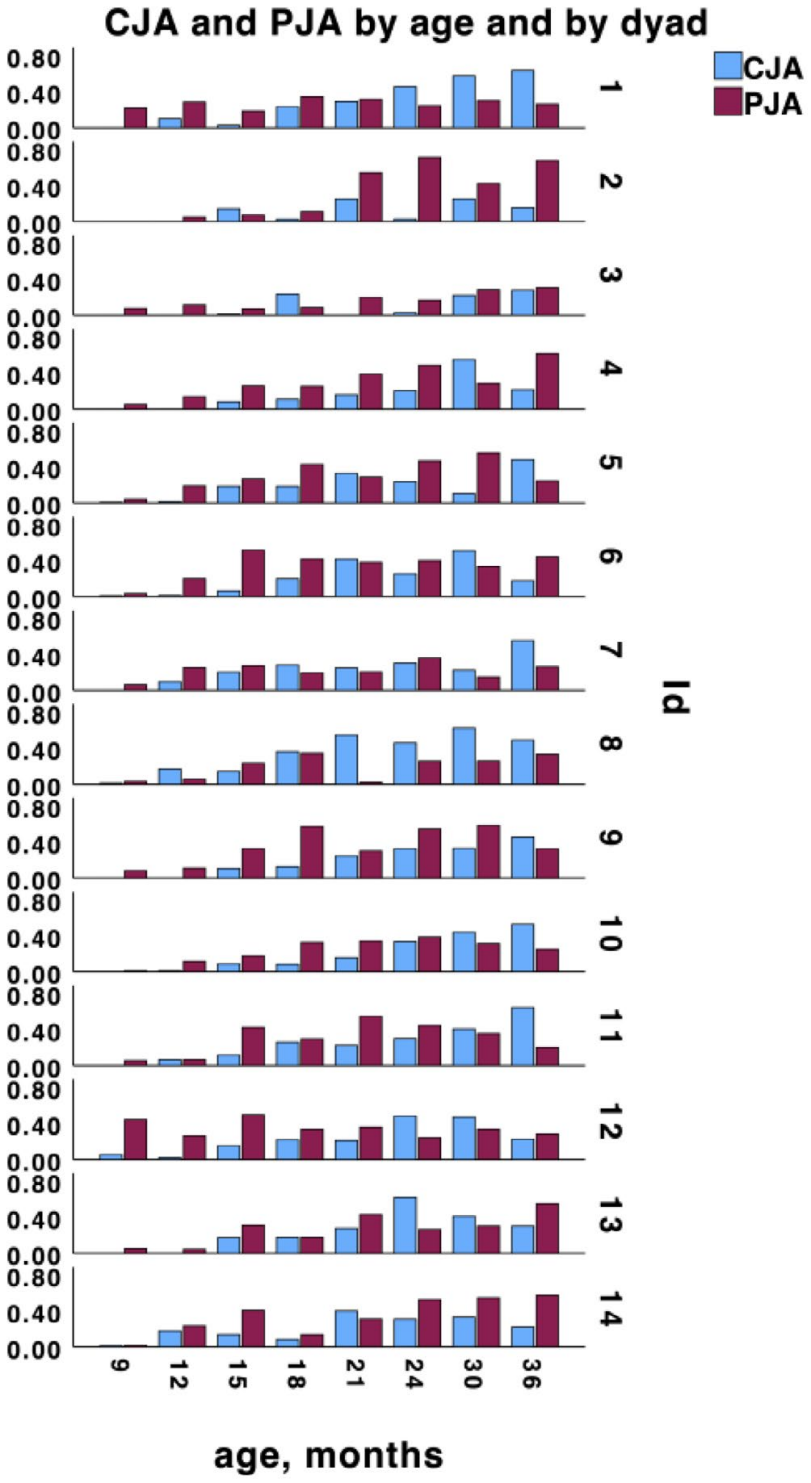
Proportion of time in CJA and PJA by dyad and age in months.

Across dyads, five children initiated JA at least once at 9 months, and in one case (Child 12) the dyad spent nearly half of the session in JA (see Figure 4). All but one parent also succeeded in establishing JA at least once at this early age, that is, the children do respond to parental JA bids. Although JA behavior might plausibly vary depending on which parent accompanies the child, no systematic differences emerged when comparing dyads with the same parent across all sessions (Children 2, 9, 10, and 12) to those in which parents alternated. For example, in the mother–child dyad of Child 8, the child initiated close to all successful JA episodes at 21 months despite being accompanied by the same parent as in earlier recordings. This underscores that variation arises not only from children's momentary state but also from fluctuations in parental attentiveness and possibly mood.

Overall, the proportion of child‐initiated JA tended to increase with age, although developmental trajectories were only linear in three dyads (1, 9, 10). By 36 months, the average proportion of time spent in JA had risen to 76%, largely driven by all but two dyads (3 and 12). At this age, seven children initiated more JA episodes than their parents (Children 1, 5, 7, 8, 9, 10, and 11), reflecting a developmental shift toward child‐led coordination of attention.

**FIGURE 5:**
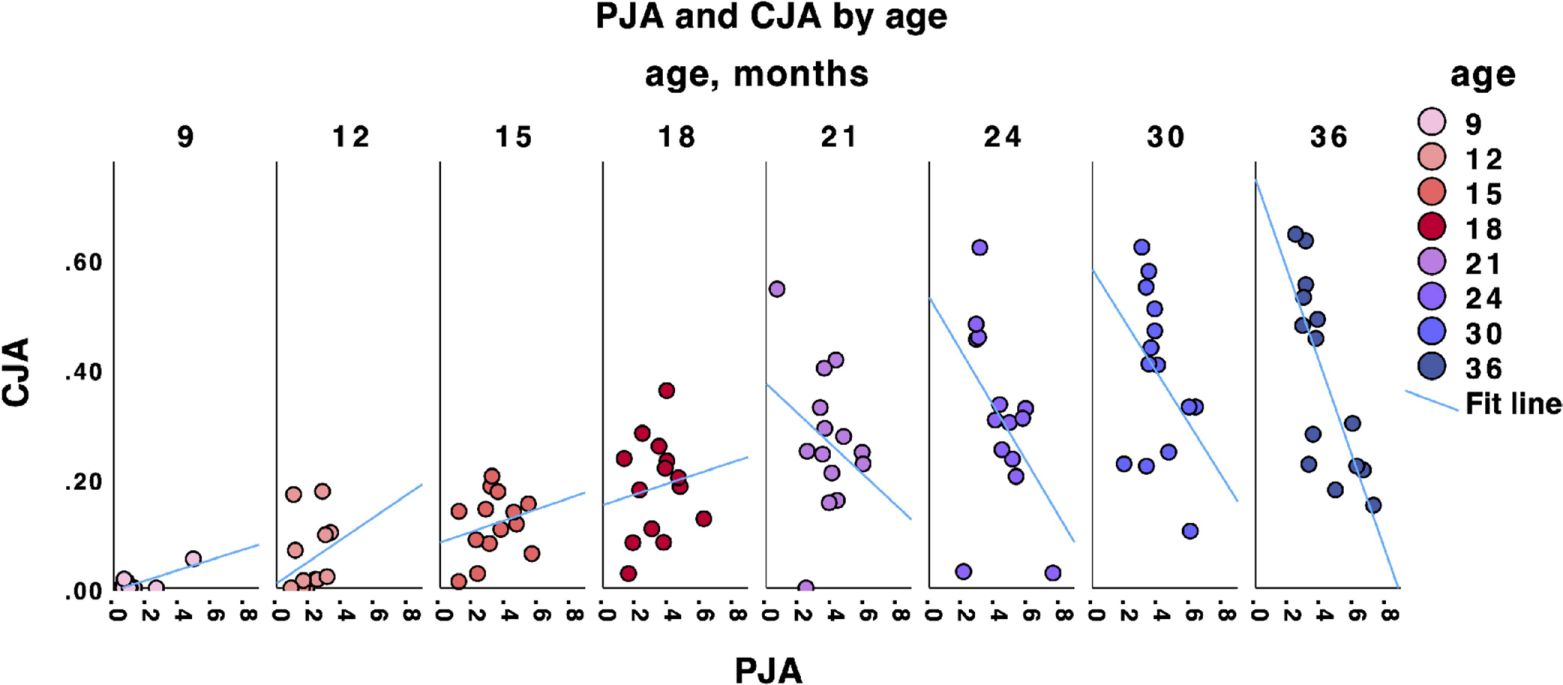
Correlation of parent and child‐initiated JA by age. An initial positive correlation at young ages gradually becomes a negative correlation at older ages.

This pattern is further illustrated in the changing relation between child‐ and parent‐initiated JA (see Figure 5). During the first four recordings (9–18 months), the association between the two was positive (Pearson correlation *r* = 0.76, *p* = 0.002 at 9 months of age). Thereafter, the correlation shifted markedly, becoming strongly negative at 36 months (Pearson correlation *r* = −0.78, *p* = 0.001).

Regarding individual differences, five children initiated joint attention at 9 months. By 12 months, nine children produced between one and twelve successful JA initiations (see Supporting Information S6). From 15 months onward, all children initiated JA at least once per session. Across subsequent recordings, initiation frequency varied considerably within individuals. For example, one child initiated 11 JA sequences at 18 months but none at 21 months, despite being accompanied by the same parent at both sessions (Child 3; see Supporting Information S1c). JA duration was generally shorter for children than for parents; however, several children reached durations comparable to or exceeding those of their parents at different ages. This occurred at 9 months (Children 5, 6, 8, and 14), 12 months (Children 1 and 10), 15 months (Children 2, 3, and 4), 18 months (Children 7 and 12), 21 months (Child 9), and 30 months (Child 11).

### 3.2 Behaviors Predicting Triadic JA Over Time

To further investigate temporal relations between JA and other behaviors, we used time‐window sequential analysis and calculated odds ratios for variables that precede JA for dyads and sessions. Thus, we aimed to investigate whether the presence of a particular behavior *X* increases the probability that JA will be initiated in the next second. In this analysis, we included all types of variables, both specific behaviors and composites such as Shared Gaze object (SGo; see Table 2). The analysis shows the variables that predict JA in the following second, and thus may be part of initiating JA episodes. If the odds ratio is larger than 1, a variable is predictive of JA, and if the odds ratio is smaller than 1, it is more likely that JA is not present the following second after behavior *X* (Yoder and Tapp 2004; Chorney et al. 2010). Table 4 presents the variables for which the 95% CI for odds ratio does not include 1.

**TABLE 4:** Observed behaviors predicting JA within a pre‐defined window (1 s preceding JA).

Variable	9	12	15	18	21	24	30	36
CG_object	2.9	5.6^b^	3.1^a^	3.1^a^	1.9	4.0^a^	2.4	1.6
PG_object	2.5	4.4^a^	3.2^a^	3.1^a^	2.4	3.4^a^	2.4	1.9
SGo	2.5	3.8^a^	2.4	2.3	1.6	2.7	1.7	1.5
P_Neutral	2.2	4.4^a^	3.3^a^	2.2^a^	4.0^a^	3.5^a^	3.7^a^	2.6
C_Neutral		5.7^b^	3.3^a^	3.2^a^	3.4^a^	3.1^a^	3.0^a^	1.8
Failed_JA		1.7	1.8	3.2^a^	5.8^b^	7.0^b^	7.8^b^	6.0^b^
CGe_Action		2.3	2.5	2.1	1.9	2.1	2.2	1.4
PG_child	1.9	2.6	3.0^a^	2.3	2.4	2.8	2.0	
CG_away		2.5	3.0^a^	2.4	3.7^a^	3.4^a^	3.3^a^	1.7
PG_away		2.2	2.1	2.1	3.5^a^	3.0^a^	2.9^a^	
P_Vocal		4.0^a^	2.1	1.6	2.6	1.9	1.4	
C_Positive	2.3	2.2	1.5	1.5	1.7	2.2		
P_Positive		2.6	1.9		1.7	2.1	1.5	
PGe_Action			1.8	1.6		1.9	2.0	1.4
CT_Comfort			1.7		2.2	1.8	2.0	
PT_Comfort		1.9	2.4		1.9		1.8	
PGoPV	2.0	3.4^a^	1.8			1.8		
CGoPV		3.0^a^	1.5			1.8		
SGoPV	1.9	2.8				1.6		
C_Vocal			2.0	1.8	1.4			
P_Show/offer		2.7			1.6			
P_Deictic					2.2			
C_Handle_object		4.5^a^						
CGp		2.2						

*Note:* Yule's *Q* = (OR – 1)/(OR + 1).

^a^ Yule's *Q* > 0.5.

^b^ Yule's *Q* > 0.7.

The odds ratio of the combined data over ages illustrates the behaviors predictive of JA in the data set. The most frequent predictors are the gaze measures Child Gaze object, Parent Gaze object, and the composite Shared Gaze object. Together with Parent Mood neutral, these are predictive throughout the recording period. Child Mood neutral and Failed‐JA are strong predictors from 12 months of age. Parent vocalizations, often included in JA by definition, are predictive from 12 to 30 months of age, and strongest so at 12 moa. Child vocalizations appear at three ages, and Child Gaze parent is only predictive at 12 months of age. We also find comforting touch among the frequent predictors, potentially including many attention‐getting touches (Koester and Lahti‐Harper 2010; Loots and Devisé 2003; Gabouer et al. 2020).

#### 3.2.1 Individual Differences in Predictors of JA Per Dyad

Across dyads, aggregated over age, two variables consistently predicted joint attention for all 14 children: Child Gaze away and Failed‐JA (see Supporting Information S7). All gaze‐related behaviors, with the exception of Child Gaze parent, were predictive in most dyads (11–12 of 14). The only gesture predicting JA in a majority of dyads was Child Gesture action (10 of 14). Neutral affect was also frequently predictive: Child Mood neutral in 11 dyads and Parent Mood neutral in 12. Parent Mood positive predicted JA in 7 dyads. Parent and Child Touch comfort were predictive in 8 dyads; in 5 of these, both partners contributed comforting touch, whereas in the remaining dyads the effect was driven by only one partner. Other behaviors were predictive within individual dyads but did not generalize across the sample.

Because behaviors were aggregated across time, these analyses do not indicate whether parent–child correspondences occurred within the same recording session. Odds ratio analyses by dyad and age (see Supporting Information S8) showed that at 9 months, 11 of 14 dyads displayed at least some overlapping behaviors preceding JA. These overlaps were predominantly gaze‐related, most commonly parent and child gaze at object and shared gaze at object. Other jointly predictive behaviors included action gestures, positive affect, comforting touch, and, in one dyad (Dyad 13), vocalization.

Across development, dyads differed substantially in the degree of behavioral overlap within sessions. Some dyads showed few shared predictors (Dyads 2, 6, 8, 13, and 14), whereas others exhibited multiple overlapping and recurring predictive behaviors (Dyads 3, 4, 5, 9, 11, and 12). Failed joint attention predicted subsequent successful JA in all dyads, particularly between 21 and 36 months. This pattern aligns with the negative correlation between child‐ and parent‐initiated successful JA from 21 months onward (Figure 5). Gesture types such as iconic, emphatic, emblematic, and deictic pointing were rare as precursors to JA before 21 months, with only isolated instances (e.g., one emblem at 9 months in Dyad 8; a show/offer at 12 months in Dyad 11; an emphatic gesture at 15 months in Dyad 7). Several gesture types did not occur as predictors in many dyads: five dyads showed no deictic gestures predicting JA (1, 2, 4, 5, 14), ten showed no iconic gestures (1, 3, 4, 5, 7, 10, 11, 12, 13, 14), and emphatic gestures were present in only five dyads (1, 5, 7, 12, 14).

The number of predictive behaviors per dyad ranged from 0 to 6 and was relatively evenly distributed across dyads and ages (see Supporting Information S8). The only consistent pattern was a higher number of predictive behaviors for both parent and child at 9 months. No systematic differences were observed depending on whether the mother or father attended the recording, either in the number or type of predictors (Supporting Information S8).

## 4 Discussion

### 4.1 How Much Time to Dyads Spend in Triadic Joint Attention Over Dyads and Time

The proportion of time children spent in triadic JA increased steadily from 7.5% at 9 months of age to 76% at 36 months (Figure 2). In comparison, Bakeman and Adamson (1984) reported an increase in coordinated JA from 2.3% to 26.6% between 6 and 18 months in a longitudinal study of 28 children observed across four sessions in a home environment. In the present data, JA constituted 47% at 18 months of age (Figure 2), which is markedly higher. The lower JA proportions reported by Bakeman and Adamson (1984) may be attributable to contextual factors—namely, home versus studio settings. However, both studies employed similar instructions to parents, comparable session durations (approximately 10 min), and involved children from similar socioeconomic backgrounds. A plausible interpretation is that parent–child interactional patterns have changed significantly since the early 1980s, or that cross‐national differences—such as those between Sweden and the United States—account for the variation in JA frequency.

Turning to non‐WEIRD samples, Mastin and Vogt (2016) found that coordinated JA constituted 17%–18% of interaction time in a sample of rural and urban 14‐month‐old Mozambican children. In a study conducted in a rural Nigerian village, Childers et al. (2007) reported that children aged 14–31 months spent approximately 26% of the time in coordinated JA. These findings contrast sharply with those of the present study, even accounting for the fact that most previous research has focused on children between 12 and 24 months of age. Cultural differences in interactional norms, as well as different recording contexts, may partly explain the discrepancy but varying annotation procedures could be involved as well. Interrater agreements, while frequently reported in studies, are typically not performed between studies but could contain clues to our different findings.

The large variation in amount of JA between the present one and earlier rapports, adds to the uncertainty related to JA as a concept. What is it we measure and in what way do we believe this composition of behaviors adds to language development? Interaction is difficult to monitor, there are many behaviors involved, at least two people's actions to consider. Timing, initiation, apart from fluctuating attention and mood, all play their parts. JA having been singled out as a construct which in particular relates to a child's language progress could depend on it being a behavior possible to study: we can detect when parent and child focus on an object, that they comment on that object, and finally exchange glances indicating that they both are aware of the shared attention. But by searching where there is light, we risk overlooking other fruitful behaviors or causes related to language. One way to start on another path, still relating to JA as a concept, is to investigate the behaviors leading up to successful JA (see Section 4.2). Another way is to start investigating the difference between dyads in the use of JA (Section 4.1.1).

#### 4.1.1 Individual Differences in Child‐ and Parent‐Initiated JA Per Dyad

A persistent challenge in studies of JA is the substantial variability in how it is defined and operationalized. In the present study, based on definitions of triadic JA (Tomasello and Todd 1983; Tomasello and Farrar 1986), and including facial expression/mood and touch together with the more common gaze, vocalization, and gesture, we observed considerable variation both within and between dyads during the first 30 months of life (see Figure 4). Such variability may reflect differences in daily routines, mood, or engagement in other forms of interaction, and is likely present in other datasets as well. It may also relate to the age metrics typically used in first language acquisition research. Although developmental variability is widely acknowledged, participants are usually grouped by chronological age in months. In practice, however, age ranges within these groups often vary due to logistical constraints and participant availability.

In the present sample, the range in age (measured in days) varied substantially, with differences spanning 21 to 66 days across the dataset (see Supporting Information S1c). In group‐level analyses of time spent in JA, this variation may not substantially affect overall outcomes, although specific increases in JA frequency could be sensitive to such differences. When examining individual dyads' engagement in JA over time—separately for child‐ and parent‐initiated JA (see Figure 3)—variation in the intervals between recordings becomes more relevant, as some dyads had shorter and others longer gaps between sessions.

However, inspection of individual cases suggests that age deviation alone does not explain the observed patterns. For example, Child 3 showed a gradual and relatively flat developmental trajectory, with no child‐initiated JA until 15 months of age. This dyad's recordings were consistently close to the target ages, indicating that the delayed increase in JA was not attributable to uneven recording intervals. The largest deviations from target age occurred for Child 1 and Child 10 at the 9‐month recording (+53 and + 25 days, respectively; see Supporting Information S1b). Yet, as shown in Figure 4, these children did not display more JA than their peers. Although based on a small sample, this suggests that deviations from target age are less influential than dyad‐specific interactional patterns—and possibly parent and child personality traits—in shaping JA development.

When correlating child‐initiated and parent‐initiated JA across dyads and ages, a positive association in the earlier months shifted to a negative association from 21 months onward. One interpretation is that as children become increasingly skilled at initiating JA, they assume a more leading role in coordinating shared attention, while parents correspondingly reduce their initiation attempts. Alternatively, both partners may increasingly attempt to guide the interaction, resulting in competing bids for attention. Contextual factors may also contribute to this pattern. Parents, aware of being recorded, may seek to highlight their child's abilities, at times prioritizing their own agenda over the child's. This issue is difficult to resolve, as home recordings are likewise affected by camera presence. A larger sample would help clarify whether the relation between parent and child initiation warrants further investigation.

A more detailed examination of dyadic similarities and differences across age (see Supporting Information S7) shows that parent‐initiated JA sequences were generally longer in duration and more frequent in the earliest recordings. The shorter duration of child‐initiated JA at early ages likely reflects children's tendency to disengage from the interaction they initiated in order to explore nearby objects. In contrast, parent‐initiated JA may be prolonged by the parent's sustained engagement. As children mature, they become both more capable of and more interested in maintaining engagement for extended periods.

Variation was evident both within dyads across sessions and between dyads overall. A child or parent who initiated many JA episodes in one recording might initiate very few—or none—in the next. Overall, aside from the general developmental trend toward more frequent and longer JA sequences from approximately 21–24 months—when parent and child become more equal in initiation and duration—the findings underscore the many factors that cannot be controlled in such analyses. Variables such as mood, fatigue, and events preceding or following the recording likely influence the interaction captured in any given session.

While this variability characterizes all social interaction, it complicates attempts to link interactional patterns to language acquisition in early childhood using relatively simple measures such as frequency and duration of JA within limited recording periods. Moreover, dyads do not appear to exhibit fixed behavioral patterns. Clear and stable predictors of JA emerge at the group level, yet these patterns largely dissolve when examined at the dyad level, with the exception of gaze toward object in its various forms (SGo, CGoPV, SGoPV, PGoPV). Home‐based recordings might yield different distributions of behaviors, potentially including more frequent child vocalizations and child gaze toward parent—attention‐getting strategies that may have been less necessary in the structured studio environment.

Taken together, these findings suggest that joint attention is shaped by continuously shifting configurations of child abilities, parental responses, and contextual affordances. The contrast between relatively robust group‐level tendencies and substantial dyad‐level variability points to development as a process of ongoing reorganization rather than the linear accumulation of stable skills. In this sense, the observed variability may reflect the adaptive flexibility of the dyadic system, where stable patterns emerge over time from locally variable interactions (e.g., Thelen and Smith 1994).

### 4.2 Predictors of Joint Attention Across Development

In the earliest recordings, joint attention was primarily predicted by parental behaviors, including gaze toward objects with or without verbalization and shared gaze combined with speech, supporting earlier findings that adults more frequently initiate JA than children (Bakeman and Adamson 1984). The only early non‐gaze predictor was the parent's show/offer gesture at 12 months, together with positive affect in both partners and, from 12 months onward, neutral affect. These findings align with previous work emphasizing parental responsiveness in the emergence of JA (Rollins 2003; Yu and Smith 2013).

From 12 months onward, child behaviors increasingly predicted JA, including gaze at objects, action gestures, and, from 15 months, vocalizations. Neutral mood in both partners remained a strong predictor, indicating that triadic engagement is facilitated in emotionally stable interactional contexts.

Failed joint attention and gaze‐away behaviors emerged as consistent predictors of subsequent JA from 12 months, suggesting that brief disengagements often precede successful re‐engagement. Concurrent predictive effects of parental action and show/offer gestures further indicate increased parental efforts to regain the child's attention when it is directed elsewhere.

Deictic pointing, often regarded as central to triadic JA, appeared as a predictor only at 21 months, suggesting that in contexts of close physical proximity and limited child mobility, object manipulation and showing may be more functional than pointing. Facial expression/mood remained predictive across development, with child positive affect predicting JA from 9 months and neutral affect in both partners becoming increasingly important thereafter, highlighting the role of emotional regulation in sustained joint engagement. Given the limited prior research linking facial expression to early language development, this consistent association warrants particular attention.

Touch, a rarely examined modality in JA research (see, however, Gabouer et al. 2020), consistently predicted JA from 12 to 30 months, primarily in the form of comforting touch, underscoring the facilitative role of physical proximity. Across all ages, gaze at the object was the strongest and most consistent predictor of JA. Parental gaze at the child was predictive at most ages, whereas child gaze at the parent played a more limited role, suggesting that parents typically drive object‐centered interactions in this context.

Finally, vocal behaviors predicted JA mainly for parents across development, while child vocalizations were predictive only between 15 and 21 months. Overall, shared gaze at objects, object‐directed action, physical proximity, and emotionally stable interactional contexts emerged as the most robust conditions for triadic joint attention.

#### 4.2.1 Individual Differences in Predictors of JA Per Dyad

It is noteworthy that some of the strongest predictors of joint attention in this sample—both in the age‐based analyses (Table 4) and at the dyad level (Supporting Information S7)—were behaviors that signal *non‐interaction*, specifically Failed‐JA and Child Gaze away. This pattern suggests that JA often emerges out of moments of disengagement, when either the parent or the child intensifies their efforts to re‐establish shared attention. Such situations may arise when the child is temporarily absorbed in independent activity (e.g., object exploration or looking away from the interaction).

Parent Gaze away was also predictive in 12 of the 14 dyads. One interpretation is that children attempt to re‐engage a parent whose attention has shifted. A more plausible explanation, however, is that parents scan the environment to locate objects or events that can serve as anchors for re‐initiating joint engagement. Together, these findings suggest that behaviors characterizing a dyad's typical interactional repertoire—whether interactive or disengaged—may function as precursors to JA within that dyad.

Among interactive behaviors, Gaze at object emerged as a robust predictor across dyads, likely due to its high frequency. Parent Gaze at child was also consistently predictive, aligning with parents' dominant role in initiating JA for much of the study period (see Figure 3). In contrast, Child Gaze at parent and Child gesture show/offer were predictive only sporadically. This pattern suggests that when children initiate JA, they may rely on different modalities than parents. Indeed, child‐initiated JA was more commonly preceded by Gesture action (10 of 14 dyads) and Comforting touch (8 of 14 dyads).

At the dyad level, however, we also observed notable overlap between parent and child predictors. Shared predictive behaviors included Gaze at object (10 dyads), Mood neutral (9 dyads), Touch comfort (5 dyads), Gesture action (5 dyads), and Mood positive (3 dyads), alongside a small number of isolated cases. One dyad (A09) showed extensive overlap across five predictive behaviors, whereas most dyads shared only Gaze away (12 dyads) and Shared gaze at object (11 dyads). These findings point to substantial individual variation in how JA is achieved, even when global patterns appear similar.

Overall, dyad‐level predictors of JA closely mirror the odds ratios computed across age, while also revealing pronounced individual differences. To further examine this variability, we conducted odds ratio analyses by dyad and age (Supporting Information S8). Most behaviors occurred at least occasionally across dyads, but gaze‐related behaviors were the most consistently predictive, particularly during the early recording session (9 months). This suggests that gaze may serve as a primary cue for establishing JA early in development, whereas vocalizations and other modalities gain importance as children become more securely embedded in the interactional framework through increased linguistic competence and interactional experience.

One dyad (A02) deviated notably from this pattern, showing few shared predictive behaviors (limited to Gaze away at 18 months and Positive mood at 21 months) and no shared gaze‐related predictors. This dyad also exhibited very few successful JA episodes between 9 and 21 months (Figure 4), which likely accounts for the limited set of predictors. In contrast, another dyad (A03) also showed few successful JA episodes overall but displayed strong overlap in parent and child predictors, including multiple shared gaze behaviors (Supporting Information S8). This contrast indicates that interactional synchrony alone does not straightforwardly predict JA success or failure. Predicting language based on these interactional differences might give us a clue to their potential importance.

While early gestures like the deictic pointing repeatedly have been connected to JA (e.g., Colonnesi et al. 2010), there were few gestures that predicted JA in the data. Comparing the dyads over age, interactional gestures like deictic pointing, show/offer gestures, and emblems appeared more broadly from 21 months of age and onwards (see Supporting Information S8). A few instances of early gesture use were seen in an emblem at 12 months of age (A08), an emphatic predictor (A07) and a show/offer (A11), both at 15 months of age. One possible interpretation is that when such gestures occur at younger ages, they are embedded within an already established JA sequence, whose initiation is instead predicted by other behaviors, such as gaze, vocalizations, or touch. At later ages, when JA becomes frequent and entire sessions may consist of successive JA episodes separated by brief interruptions, gestures emerge as predictors within a dense interactional web. In this phase, interruptions between JA episodes are better characterized as pauses rather than periods of non‐interaction.

Finally, the frequent predictive role of Failed‐JA, particularly from 21 months onward, suggests increased persistence in pursuing engagement at this age. Both partners appear more attuned to the interactional context and more willing to repeatedly attempt re‐engagement, often successfully. Familiarity with the laboratory setting and procedures—returning every 3 months and encountering both new and familiar toys—may further support these increasingly effective re‐engagement strategies.

Apart from Failed JA, there is no pattern in the predictive behaviors when looking at the dyads separately and over age, and considering whether it was the mother or father present (Supporting Information S8: Key c.). This make us conclude that predictive behaviors varies both within and between dyads, neither child or parent appear to perform behaviors that are stable neither in the short time span (appr. 10 min) nor in the longer (9–36 months).

Concluding the investigation of individual similarities and differences over age for the 14 dyads we can state that the variation in predictive behaviors, within a dyad and between them, is great. While shared gaze at object, action‐gestures, and comforting touch were predictive when aggregating the data, these patterns are less clear when breaking it up in dyads and over age. One of the strongest predictive behaviors of JA, Action Gestures, were not present as predictive behaviors at all in five of the dyads. This indicate that Comforting Touch, Child gaze at object, and Neutral mood, are our strongest clues to what precedes a JA through 9–36 months of age. We can also conclude that all gestures but show/offer are infrequent, possibly appearing more often once a JA has begun. The behavioral difference both within and between dyads is also a clue to future studies: if the aim is to understand language acquisition, we need to look at individual children, preferably many. The composition of behaviors leading to interaction and subsequent language progress might not be found in specific behavioral composites but rather in amount of practice of whatever interactional behavior the dyad prefers.

## 5 Conclusions

The present study found triadic joint attention to be highly frequent, accounting for 76% of interaction time at 3 years of age. However, substantial individual variation was observed among the 14 dyads, indicating that individual differences should be taken into account when using joint attention as a predictor of language acquisition. Until we know more of individual children's trajectories, it remains possible that joint attention is but one of many interactional forms that may—or may not—contribute to a child's language acquisition process.

At the group level, gaze directed at the object, close physical proximity between interactants, neutral affect, and action‐based gestures (e.g., moving a toy car along the floor) were significant predictors of successful joint attention. In contrast, analyses of individual dyadic trajectories revealed that only the negative predictors Failed joint attention and Gaze away consistently preceded successful joint attention across the sample. This pattern may suggest that both parents and children actively attempt to re‐establish mutual engagement following moments of disengagement. Differences between dyads may reflect the use of distinct interactional strategies; nevertheless, within the constrained recording context, certain behaviors—such as Gaze at object—were highly likely to occur across dyads, whereas gestures such as Emphatic, Iconic, Deictic, and Emblems were infrequent or not present at all as predictors. The individual differences between dyads further indicate that more fine‐grained interactional patterns—and possibly amount of interaction—might be a better way to increase our understanding of language acquisition than collapsing children into groups based on age.

The methodological approach, time‐window sequential analysis, proved effective in identifying both group‐level patterns and the diversity between and within dyads' interactional styles. Moreover, analyses based on age measured in days indicated that age differences—even up to more than 1 month—played a minor role relative to this variability in dyadic interactional styles.

## 6 Limitations

A limitation of the present study lies in the quantification approach. Continuous behaviors during parent–child interactions—such as gaze, gesture, vocalization, touch, and facial expressions—were measured in terms of duration and temporal overlap using one‐second intervals. While this method facilitated manageable analyses, it obscured the precise deployment of specific modalities within individual JA episodes. Furthermore, some behaviors (e.g., rapid gaze shifts) are brief yet communicatively significant, whereas others (e.g., sustained touch or neutral expressions) are longer but less informative. These differences were not captured, potentially affecting interpretations of behavioral importance. Different behaviors duration and clarity might also have affected what was considered an initial behavior of JA. While gaze toward the other, verbalizations, or touch as attention‐getter are easy to interpret as initiative moves, subtle changes in body posture and facial expressions, which might be clear to the interlocutor and function as a response‐trigger, are harder to identify. This could lead to behaviors like gaze and verbalizations being overrepresented as initiators of JA. If this is the case, predictions based on the second before a JA begins will be flawed as well. An additional limitation is the small sample size; findings should therefore be regarded as descriptive observations within a unique longitudinal dataset rather than as generalizable population estimates.

## Author Contributions

Tove Nilsson Gerholm: Conceptualization; Data curation; Funding acquisition; Investigation; Methodology; Project administration; Resources; Supervision; Validation; Writing – original draft; Writing – review and editing. Petter Kallioinen: Formal Analysis; Investigation; Methodology; Visualization; Writing – review and editing. Tatjana von Rosen: Formal analysis; Methodology; Validation; Visualization; Writing – original draft; Writing – review and editing.

## Funding

Research reported in this study was funded by The Swedish Research Council (2018‐01135, 2019–2022) and Marcus and Amalia Wallenberg Foundation (2013–2017, 2011.007). PI Tove Nilsson Gerholm.

## Ethics Statement

The MINT‐project, of which this study was a part, was approved by the Regional Ethics Committee in Stockholm (DNR: 2011/955‐31/1; DNR: 2020/03578‐10/2) and parents received written information on their right to withdraw their consent at any time. The participant information was handled according to the General Data Protection Regulation (GDPR 2016/679). All participants have been anonymized.

## Conflicts of Interest

The authors declare no conflicts of interest.

## Supporting information


**Table S1a.** Background information 2 years of age.
**Table S1b.** Recording information: age at recording calculated as +/− days from respective target age 9 months, 12 months, etc.
**Table S1c.** Recording information: accompanying parent per child and age.
**Table S2.** Transcription and annotation guide.
**Illustrations S3.** Annotation of joint attention and failed joint attention.
**Illustrations S4.** Interrater agreement.
**Code S5.** Code used for extracting data from ELAN.
**Table S6.** Individual differences in frequency and length of JA per dyad and age.
**Table S7.** Individual differences in predictors of JA per dyad.
**Table S8.** Individual differences in predictors of JA per dyad and age.

## Data Availability

The data cannot be shared according to GDPR and ethics approval, as they contain sensitive information on individual children and families. Specifics on recordings, transcription, and annotation procedures, as well as inquiries about methodology and analysis, can be directed to the first author.
