# Supplementary material for: Developmental Trajectories and Sequential Analysis of Triadic Joint Attention
Source: Scand J Psychol. 2026 Mar 29;67(4):1142–56. doi: 10.1111/sjop.70096 (PMC13352546; doi:10.1111/sjop.70096)
Supplement: Supplementary file 1 — Table S1a. Background information 2 years of age. Table S1b. Recording information: age at recording calculated as +/− days from respective target age 9 months, 12 months, etc. Table S1c. Recording information: accompanying parent per child and age. Table S2. Transcription and annotation guide. Illustrations S3. Annotation of joint attention and failed joint attention. Illustrations S4. Interrater agreement. Code S5. Code used for extracting data from ELAN. Table S6. Individual differences in frequency and length of JA per dyad and age. Table S7. Individual differences in predictors of JA per dyad. Table S8. Individual differences in predictors of JA per dyad and age. [file SJOP-67-1142-s001.docx]

**Supplementary Material**: **Developmental Trajectories and Sequential Analysis of Triadic Joint Attention**

**Supplementary material S1a-c: Participant background information, Recording information, and Information on accompanying parent**

**S1a: Background information 2 years of age**

| **Child sex** | **Additional language** | **Education** | | **Siblings** | **Languages other than LI [blinded for review]** | **Family situation** | **Income (1=lower spectra; 2=middle spectra; 3=higher spectra)** | |
| --- | --- | --- | --- | --- | --- | --- | --- | --- |
|  |  | **Parent1** | **Parent2** | **First child = 0; Older siblings = 1** |  | **Living with both parents=1** | **Parent1** | **Parent 2** |
| A01_Boy | 1 | University | Highschool | 0 | [Blinded for review] | 1 | 1 | 3 |
| A02_Boy | 0 | University | University | 1 |  | 1 | 2 | 2 |
| A03_Girl | 0 | University | University | 0 |  | 1 | 3 | 3 |
| A04_Boy | 0 | University | University | 1 |  | 1 | 2 | 2 |
| A05_Girl | 0 | University | University | 0 |  | 1 | 2 | 2 |
| A06_Boy | 0 | University | University | 0 |  | 1 | 2 | 2 |
| A07_Girl | 0 | University | University | 0 |  | 1 | 2 | 2 |
| A08_Girl | 0 | University | Vocational education | 0 |  | 1 | 1 | 2 |
| A09_Boy | 0 | Highschool | Vocational education | 1 |  | 1 | 2 | 3 |
| A10_Boy | 0 | Highschool | Highschool | 1 |  | 1 | 2 | 2 |
| A11_Girl | 0 | University | University | 1 |  | 1 | 2 | 2 |
| A12_Boy | 0 | University | University | 1 |  | 1 | 2 | 2 |
| A13_Girl | 0 | University | University | 1 |  | 1 | 3 | 3 |
| A14_Girl | 0 | University | University | 0 |  | 1 | 2 | 3 |

**S1b: Recording information: age at recording calculated as +/- days from respective target age 9 months, 12 months, etc.**

| **Child sex** | **9 moa** | **12 moa** | **15 moa** | **18 moa** | **21 moa** | **24 moa** | **30 moa** | **36 moa** |
| --- | --- | --- | --- | --- | --- | --- | --- | --- |
| A01_Boy | +53 days | +/- 0 | -3 days | +32 days | +6 days | -2 days | -5 days | -2 days |
| A02_Boy | -11 days | -5 days | -6 days | +11 days | -3 days | -4 days | -5 days | -10 days |
| A03_Girl | -8 days | +5 days | +4 days | -7 days | -9 days | +4 days | +/-0 | +7days |
| A04_Boy | -1 day | +5 days | +4 days | +5 days | +/-0 | +20 days | -4 days | +9 days |
| A05_Girl | +5 days | +3 days | +2 days | -4 days | -2 days | -6 days | +2 days | -4 days |
| A01_Boy | -4 days | +2 days | +10 days | +13 days | +2 days | +18 days | -4 days | +9 days |
| A07_Girl | +1 day | +6 days | +2 days | -9 days | -12 days | -14 days | +8 days | -16 days |
| A08_Girl | +7 days | -4 days | -1 day | +5 days | +26 days | -1 day | -3 days | +2 days |
| A09_Boy | +5 days | +4 days | -3 days | -4 days | +8 days | +9 days | +8 days | +4 days |
| A10_Boy | +25 days | -5 days | -7 days | +6 days | +9 days | +2 days | -4 days | -7 days |
| A11_Girl | +6 days | +3 days | -4 days | +8 days | +8 days | -8 days | -9 days | -9 days |
| A12_Boy | -13 days | -12 days | -11 days | -11 days | -12 days | -6 days | -21 days | -15 days |
| A13_Girl | +/-0 | +5 days | -6 days | -1 day | +7 days | +4 days | -6 days | -11 days |
| A14_Girl | +11 days | +10 days | -6 days | -1 day | +1 day | +3 days | +1 day | +5 days |
|  |  |  |  |  |  |  |  |  |
|  | 9 moa | 12 moa | 15 moa | 18 moa | 21 moa | 24 moa | 30 moa | 36 moa |
| spread | -13 to +53 | -12 to +10 | -11 to +10 | -11 to +32 | -12 to +26 | -14 to +20 | -21 to +8 | -16 to +9 |
|  | 66 days | 22 days | 21 days | 43 days | 38 days | 34 days | 29 days | 25 days |
|  |  |  |  |  |  |  |  |  |
| Mean | 10,7 | 4,9 | 4,9 | 8,4 | 7,5 | 7,2 | 5,7 | 7,9 |

**S1c: Recording information: accompanying parent per child and age. M = Mother, F = Father.**

|  | 9 | 12 | 15 | 18 | 21 | 24 | 30 | 36 |
| --- | --- | --- | --- | --- | --- | --- | --- | --- |
| A01_Boy | M | M | M | M | M | F | M | M |
| A02_Boy | M | M | M | M | M | M | M | M |
| A03_Girl | M | F | F | F | F | F | M | M |
| A04_Boy | M | M | F | M | M | M | M | F |
| A05_Girl | M | F | F | F | M | M | M | M |
| A06_Boy | M | M | F | M | M | M | F | M |
| A07_Girl | M | M | F | F | F | M | F | M |
| A08_Girl | M | M | M | M | M | F | M | M |
| A09_Boy | M | M | M | M | M | M | M | M |
| A10_Boy | M | M | M | M | M | M | M | M |
| A11_Girl | F | F | F | F | M | F | M | M |
| A12_Boy | M | M | M | M | M | M | M | M |
| A13_Girl | F | F | F | M | F | F | M | M |
| A14_Girl | M | F | F | F | M | M | M | M |

**Supplementary material S2: Transcription and Annotation Guide**

[**https://osf.io/qj67w/overview?view_only=aab6071cf65541689df52ca57f663ad6**](https://osf.io/qj67w/overview?view_only=aab6071cf65541689df52ca57f663ad6)

**Supplementary material S3: Annotation of Joint Attention and Failed Joint Attention**

**
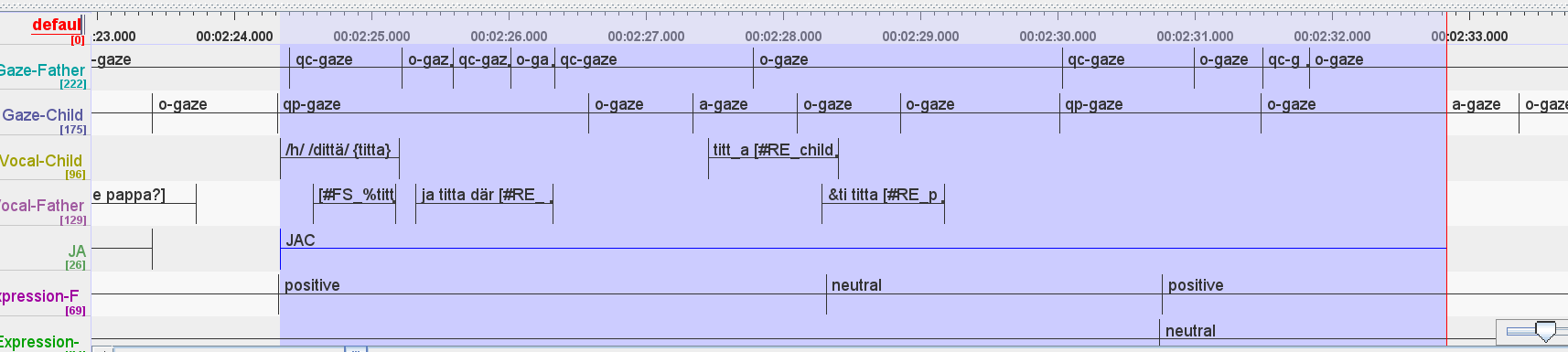
**

Above: An instance where the child is initiating a JA by looking at then parent and vocalizing. While the father’s gaze is then alternating quickly between the object and the child, the child’s gaze stays on the object for some time but then returns to the father. Meanwhile they both verbalize around the object in question. The JA ends when one of them appears to turn their attention elsewhere.


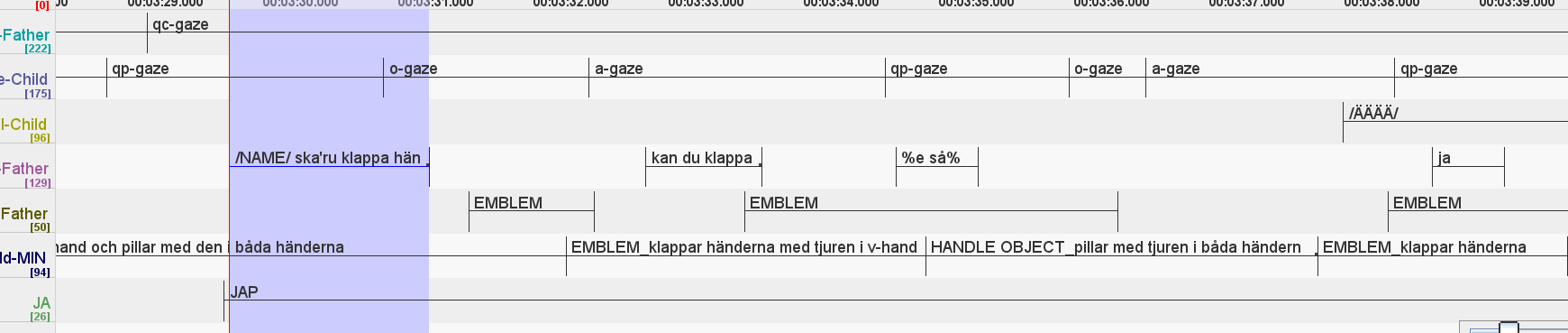


Above. A successful JA initiated by the parent and starting with a verbalization “/child name/ are you going to clap your hands”. As you can see in the two first tiers (gaze), the child and the parent are already looking at each other but the JA doesn’t commence until the parent’s verbalization some milliseconds later. This is because gaze alone will not count as a JA in the definition applied here. People close to one another are likely to monitor each other every now and then without this implying anything other than awareness of the other’s presence, or curiosity what s/he is doing, etc. For the sequence to count as a triadic JA there must be some other interactional behavior going on as well, which is what we see when the father addresses the child and the child starts clapping (Emblem).


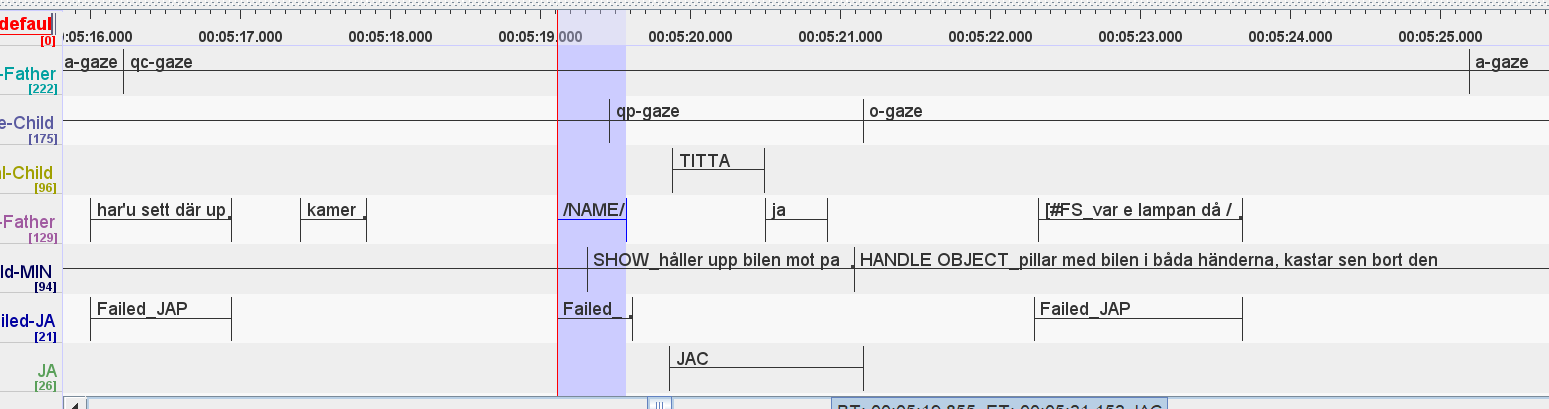


Above: Three Failed parent-initiated JA and, in between, a short successful child-initiated JA. The child is preoccupied with a toy and the father repeatedly tries to make the child attend to something else, first by “have you seen up there… the camera”, and then by calling the child’s name. The child turns to the father directly after but does not attend to the father’s suggestion to look at the lamp. Instead, he makes a show/offer-gestures and says “look” related to the toy he is occupied with. This is a successful JA since the father responds, looks at the object and utters “yes”. However, the child immediately turns back to his toy and the father continues trying to engage the child in the conversation about the lamp, unsuccessfully.

**Supplementary material S4: Interrater agreement**

Examples of how Complete, Partial, and No Agreement were applied in the Interrater agreement.


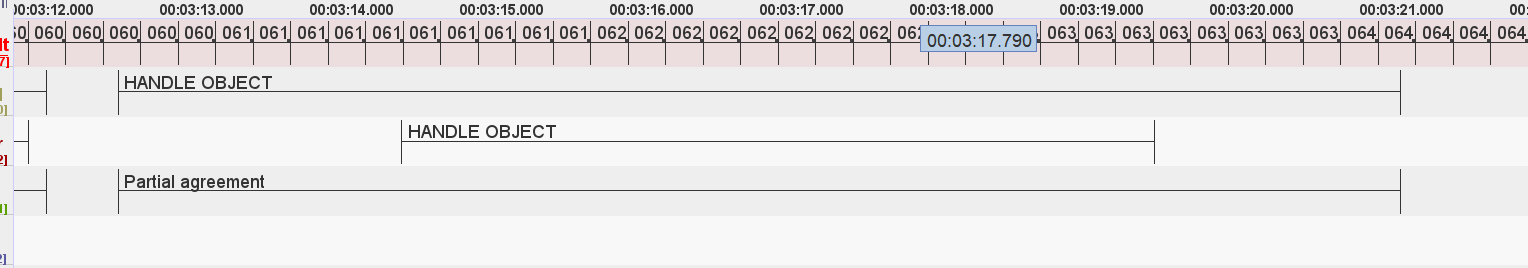
Above: Partial agreement since the time stamps differed markedly although the content was identical.

**
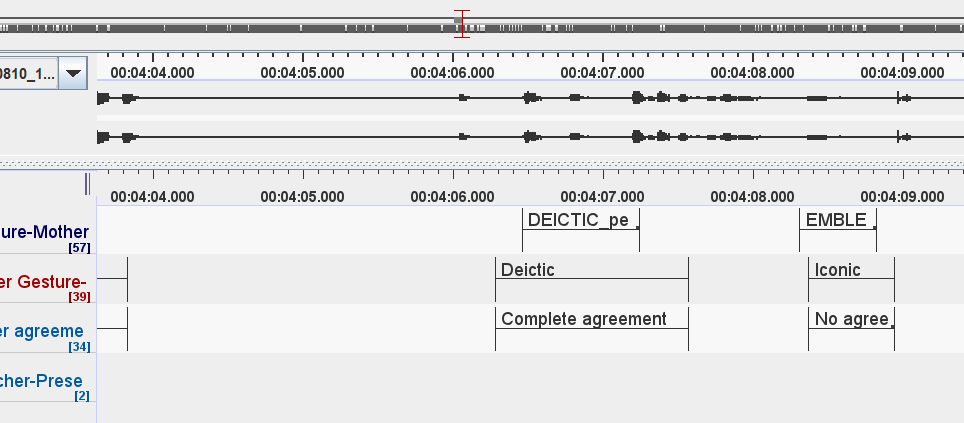
**

Above: Complete agreement for Deictic because of identical content and the small time-stamp difference will not alter analysis. No agreement since different annotators viewed the gesture – which they both noted – as being of different character.

**
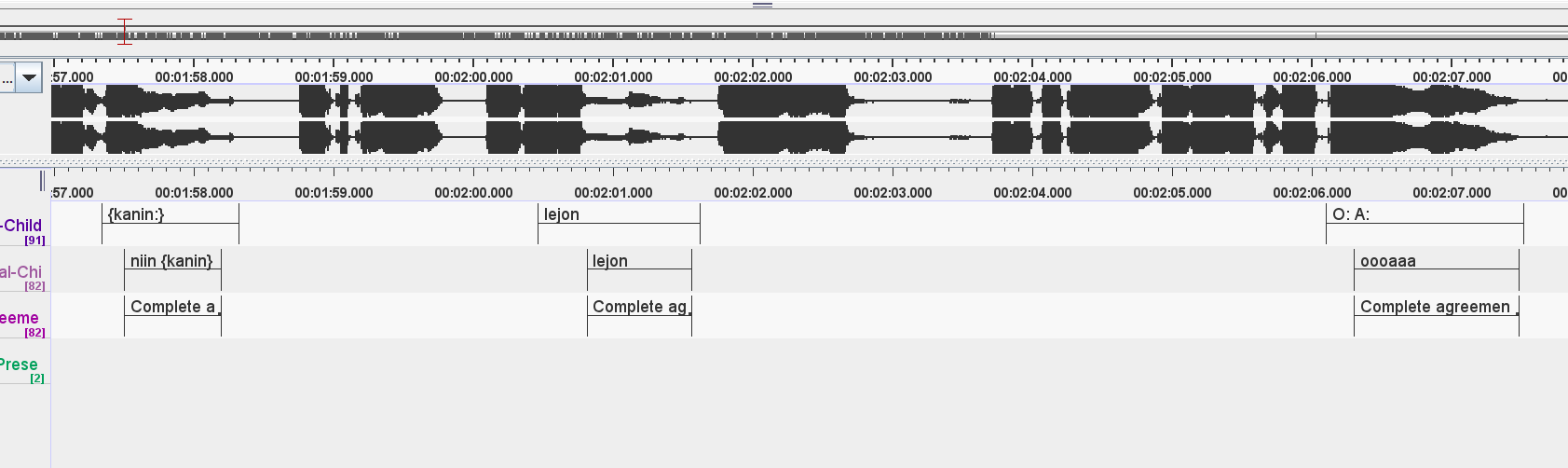
**

Above: Complete agreement even though spellings differ between the two annotators. Differing tamp-stamps for Vocalizations are due to different annotators using different zoom levels, thus seeing/hearing the beginning and end of utterance slightly differently.

**
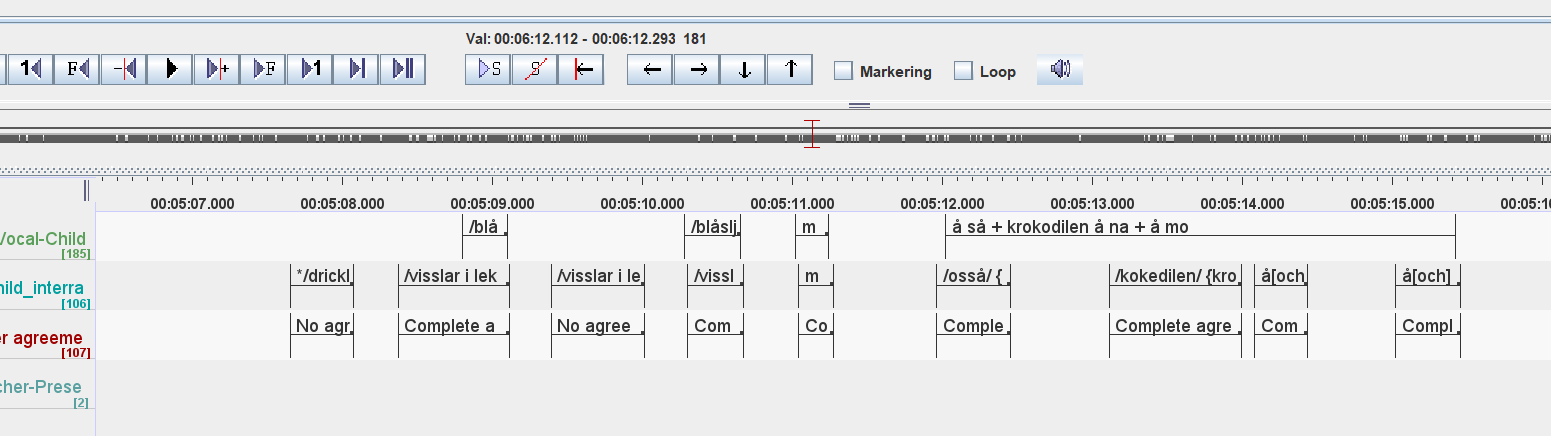
**

Above: No agreement when one annotator heard something the other one missed. Complete agreement when small time-stamp differences or when one annotator divided an utterance into (in this case) four different tags whereas the other made it one annotation. This does not alter the analyses since frequency of utterances is not used as a measure, and the time-difference created is judged as marginal (and likely to even out between annotators seen over a whole file).

**
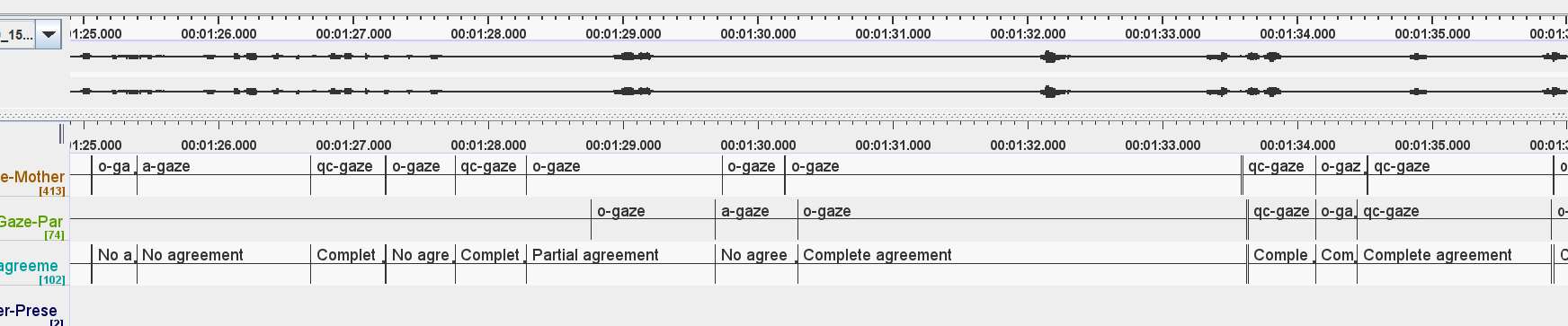
**

Above: No agreement when gaze was annotated with different tags. Complete agreement when time-stamp and content was identical. Partial agreement when time-stamps differed so that part of an annotation was considered a-gaze by one annotator and o-gaze by the other. Since Gaze and FacialExpression are always annotated, the time-stamps become more influential.

**
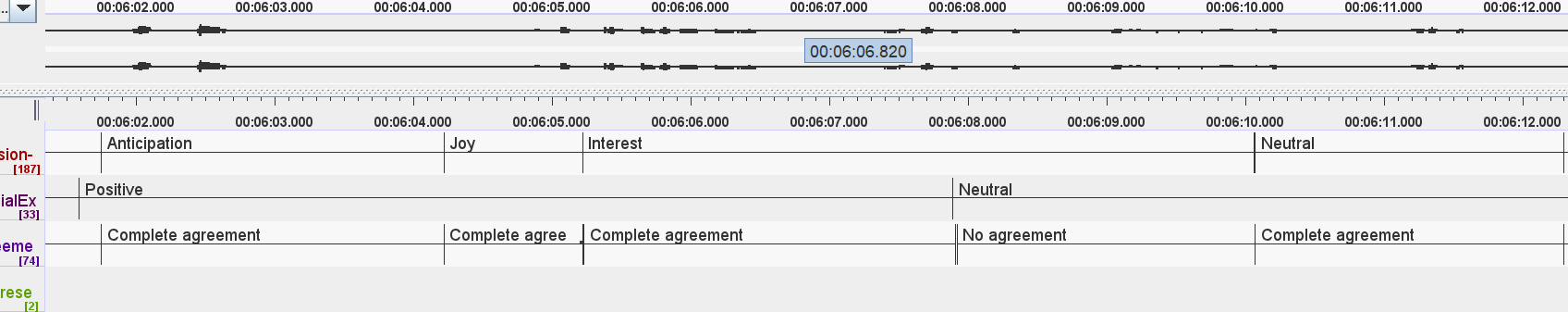
**

Above: FacialExpression using the Composites Neutral, Positive and Negative as comparison to the many variable opportunities of the transcription.

**Supplementary material S5: Code used for extracting data from ELAN**

[**https://osf.io/qj67w/overview?view_only=aab6071cf65541689df52ca57f663ad6**](https://osf.io/qj67w/overview?view_only=aab6071cf65541689df52ca57f663ad6)

**Supplementary material S6:**  **Individual differences in frequency and lenght of JA per dyad and age**

[**https://osf.io/qj67w/overview?view_only=aab6071cf65541689df52ca57f663ad6**](https://osf.io/qj67w/overview?view_only=aab6071cf65541689df52ca57f663ad6)

**Supplementary material S7:**  **Individual differences in predictors of JA per dyad**

|  | A01 | A02 | A03 | A04 | A05 | A06 | A07 | A08 | A09 | A10 | A11 | A12 | A13 | A14 |
| --- | --- | --- | --- | --- | --- | --- | --- | --- | --- | --- | --- | --- | --- | --- |
| Failed-JA | x | X | x | x | x | x | x | x | x | x | x | x | x | x |
| CGa | x | x | x | x | x | x | x | x | x | x | x | x | x | x |
| PGa | x | x | x | x | x |  | x | x | x | x | x | x | x |  |
| PGo | x |  | x | x | x | x | x |  | x | x | x | x | x | x |
| CM_neutral | x | x | x | x | x | x |  | x | x | x | x |  | x | x |
| CGo | x |  | x | x |  |  | x | x | x | x | x | x | x | x |
| SGo | x |  | x | x | x |  | x |  | x | x | x | x | x | x |
| PGc | x | x | x |  | x | x | x | x | x | x |  |  | x | x |
| PM_neutral | x |  | x | x | x | x | x |  | x | x | x | x |  | x |
| CGe_action |  |  | x | x | x | x | x | x | x | x |  | x |  | x |
| CT_comfort | x | x | x |  |  | x |  |  | x | x |  |  | x | x |
| PT_comfort |  |  |  |  | x | x |  |  | x | x | x | x | x | x |
| PM_positive |  | x | x |  |  | x |  | x | x |  |  |  | x | x |
| PGe_action | x |  | x |  |  | x | x | x | x |  |  |  |  |  |
| PGe_show/offer |  |  | x | x |  |  | x |  | x |  |  | x | x |  |
| CM_positive |  | x | x |  | x |  | x |  | x |  |  |  |  |  |
| CM_negative |  |  |  |  |  | x |  |  | x | x |  | x | x |  |
| SGoPV |  |  | x | x |  |  |  |  |  |  | x | x |  | x |
| P_Vocal |  |  | x | x |  |  |  |  |  |  | x | x | x |  |
| CGoPV |  |  | x | x |  |  |  |  |  |  | x |  | x |  |
| CGe_emblem |  | x |  |  | x |  |  | x |  |  |  |  |  |  |
| PGe_deictic |  |  |  |  |  |  | x |  |  |  | x | x |  |  |
| PGe_emblem |  |  |  |  | x |  |  |  |  |  |  | x |  |  |
| PGe_emphatic |  |  |  |  |  |  | x |  |  |  |  | x |  |  |
| PT_stimulate |  |  |  | x | x |  |  |  |  |  |  | x |  | x |
| CGp |  |  |  |  | x | x |  |  |  |  |  |  |  |  |
| PT_action |  |  |  | x |  | x |  |  |  |  |  |  |  |  |
| PM_negative |  |  |  |  | x |  |  |  | x |  |  |  |  |  |
| CGe_deictic |  |  |  |  |  |  |  | x |  |  |  |  |  |  |
| CGe_iconic |  | x |  |  |  |  |  |  |  |  |  |  |  |  |
| PGe_iconic |  |  |  |  |  |  |  |  | x |  |  |  |  |  |
| CGe_emphatic |  |  |  |  |  |  |  |  |  |  |  |  |  | x |
| C_vocal |  |  |  |  |  | x |  |  |  |  |  |  |  |  |
| CT_stimulate |  |  |  |  |  | x |  |  |  |  |  |  |  |  |
| CT_action |  |  |  |  | x |  |  |  |  |  |  |  |  |  |
| CGe_show/offer |  |  |  |  |  |  |  |  |  |  |  |  |  |  |

**Supplementary material S8:**  **Individual differences in predictors of JA per dyad and age:**

[**https://osf.io/qj67w/overview?view_only=aab6071cf65541689df52ca57f663ad6**](https://osf.io/qj67w/overview?view_only=aab6071cf65541689df52ca57f663ad6)
